# Long-Term Survival of Patients with Metastatic Non-Small-Cell Lung Cancer over Five Decades

**DOI:** 10.1155/2021/7836264

**Published:** 2021-01-12

**Authors:** Jair Bar, Damien Urban, Uri Amit, Sarit Appel, Amir Onn, Ofer Margalit, Tamar Beller, Teodor Kuznetsov, Yaacov Lawrence

**Affiliations:** ^1^Institute of Oncology, Chaim Sheba Medical Center, Tel Hashomer, Ramat Gan 5262000, Israel; ^2^Affiliated with Sackler Faculty of Medicine, Tel Aviv University, Ramat Aviv, Tel Aviv, Israel; ^3^Department of Radiation Oncology, Chaim Sheba Medical Center, Tel Hashomer, Ramat Gan 5262000, Israel; ^4^Pulmonology Institute, Chaim Sheba Medical Center, Tel Hashomer, Ramat Gan 5262000, Israel; ^5^Department of Radiation Oncology, Sidney Kimmel Medical College at Thomas Jefferson University, Philadelphia, PA, USA

## Abstract

**Objective:**

Novel therapeutics and supportive care improved outcomes for metastatic non-small-cell lung cancer (mNSCLC) patients. Major advances over the past five decades include the introduction of combination chemotherapy, small molecules targeting mutant proteins, especially EGFR, and more recently immunotherapy. We aim to document real-world long-term survival over the past five decades.

**Methods:**

Survival statistics were extracted from the Survival, Epidemiology, and End Results (SEER) database for mNSCLC patients during 1973–2015. Two- and five-year survival (2yS and 5yS) were analyzed using Kaplan–Meier and proportional hazard models.

**Results:**

The study population consisted of 280,655mNSCLC patients diagnosed during 1973–2015. Longer survival was seen in younger, female, married, Asian/Pacific Islander race, adenocarcinoma, lower grade, more recent diagnosis, higher income, and chemotherapy-treated patients. 2yS increased during the study period from 2.6% to 12.9%, and 5yS increased from 0.7% to 3.2%. 2yS of patients <50 years of age rose from 2.1% to 22.8%, and their 5yS rose from 0.7% to 6.2%. 2yS of adenocarcinoma patients improved from 2.7% to 16.2%, and their improved 5yS from 1.1% to 3.9%.

**Conclusions:**

Between 1973 and 2015, there was a dramatic improvement in long-term survival, with an approximately five-fold increase in both 2yS and 5yS. Nonetheless, absolute numbers of long-term survivors remained low, with less than 4% living 5 years. This provides a baseline to compare long-term outcomes seen in the current generation of clinical trials.

## 1. Introduction

Lung cancer is the number one cause of cancer-related death worldwide, inflicting about 1.6 million deaths annually [1]. In 2016, 158,080 lung cancer patients died in the USA alone [2].Lung cancer patients are diagnosed as metastatic disease in 40–50% of the cases [3, 4], with a median survival of less than nine months [3, 5].The most common type of lung cancer is non-small-cell lung cancer (NSCLC), comprising 85–89% of lung cancers. Significant advances in the care of metastatic NSCLC (mNSCLC) patients over the recent decades include the advent of palliative chemotherapy [5], histology-directed chemotherapy agents [6], improvements in supportive care [7], more recently targeted agents [8], and immunotherapy [9]. Modern treatments have achieved long-term survival, for instance, EGFR-mutant metastatic lung adenocarcinoma treated with erlotinib or gefitinib has a 14.6% five-year survival, and immunotherapy has achieved 13.4% five-year survival amongst patients with mixed levels of programmed death ligand-1 (PD-L1) expression [10].

Remarkably, metastatic anaplastic lymphoma kinase- (ALK-) positive NSCLC patients were reported to have a median overall survival (mOS) of around 4–5 years [11]. The European Society of Medical Oncology (ESMO) Magnitude of Clinical Benefit Scale suggests a treatment that increases the two-year survival of patients who should receive maximal support from the Oncologist community [12]. However, long-term survival of mNSCLC is rarely reported in studies and is not the focus of most retrospective database analyses.

The critical evaluation of reports of long-term survival rates of patients with novel therapies requires valid comparators; real-world data collected prior to the immunotherapy era can serve this purpose. The Surveillance, Epidemiology, and End Results (SEER) registry has been collecting data on cancer across the United States since 1973.Over time, the area covered has substantially increased, presently including 28% of the U.S. population. The population covered by the SEER registry is comparable to the general U.S. population with regard to measures of income and education, but has a higher proportion of foreign-born persons compared to the general U.S. population [13].Some previous reports of lung cancer in the SEER database focus on incidence trends [14, 15]. A more recent publication reports also on survival, however in an aggregate manner, including all types of lung cancer (small cell and NSCLC), as well as all stages [16]. Other reports focused on survival and treatment costs of squamous cell NSCLC [17] or older NSCLC patients [18]. A mathematical model predicting survival was suggested, including all stages of lung cancer, and was limited to patients diagnosed in 1998–2001 [19]. The impact of tumor size on the outcome of early stage cancer was analyzed in another report [20]. The influence of treatments on short-term (one year) survival [21] or on median survival [18] of mNSCLC was reported. Other reports focus on the factors impacting chosen treatments [22]. Another study of the SEER database queried the prognostic role of race on lung cancer survival [23], and another examined age [24], both including all stages of disease.

Unlike previous studies, we aimed to focus on advanced NSCLC, the largest group of lung cancer patients, where dramatic changes have occurred recently regarding the standard of care systemic therapy. Accordingly, we wanted to describe the changes in the relevant prognostic parameters and outcome along the 40 years leading to the current era. For this goal, we utilized the SEER database to investigate long-term survival of mNSCLC prior to the immunotherapy era. Trends over time and the impact of potential prognostic factors, including pathologic grade and subtypes, socioeconomic and social parameters, and treatments administered, were studied in a comprehensive manner. We report of one of the largest cohorts of lung cancer patients, focusing on the less well-documented long-term survival. The data presented herein can serve as a basis of comparison when evaluating long-term survivor rates of mNSCLC patients treated with immunotherapy and other novel agents.

## 2. Materials and Methods

The study utilized data from the 18 registries that comprise the SEER database (Atlanta, Connecticut, Detroit, Hawaii, Iowa, New Mexico, San Francisco-Oakland, Seattle-Puget Sound, and Utah Alaska, San Jose-Monterey, Los Angeles, Rural Georgia, Greater California, Kentucky, Louisiana, New Jersey, and Greater Georgia).

Inclusion criteria were mNSCLC diagnosed between 1973 and 2015. Primary lung cancer was identified according to the ICD-0-3 codes: C34.0 (main bronchus), C34.1 (upper lobe, lung), C34.2 (right middle lobe, lung), C34.3 (lower lobe, lung), C34.8 (overlapping lesion of the lung), and C34.9 (lung, NOS). Identified cases were divided into histology groups based on the World Health Organization classification [25]. Exclusion criteria were unknown stage, nonmetastatic disease, sarcomas, carcinoid histology, unknown histology, nonspecified malignant histology, rare or unspecific histologies (WHO ICD-O-3 morphological codes [26] 8000, 8001, 8002, 8003, 8004, 8011, 8022, 8030, 8031, 8032, 8033, 8035, 8245, 8490, 8982, 8982, and 9133), or those diagnosed only at autopsy. Data extracted included patient demographics, tumor grade, primary site, treatment category, year of diagnosis, and survival until death or last follow-up as of December 31, 2015. Treatments are reported in the SEER database as ‘chemotherapy', ‘radiotherapy,' both of these, or none; of note, the SEER database does not differentiate between ‘unknown' and ‘did not receive' for these modalities. Use of oral drugs such as tyrosine kinase inhibitors is not registered in the SEER database. Social status was estimated by the median income at 2010 of the county of each patient. Patients' missing data for a covariate were included in the study, aside from analyses that included that particular covariate.

For the purposes of descriptive (but not survival) analysis, continuous variables (e.g., age, year of diagnosis, and regional income) were converted into categorical variables. Statistical analyses were performed using the Stata statistical package, version IC 11.1 (Stata, College Station, TX). Chi-square tests were used to assess associations between categorical variables. The primary endpoint was survival, defined from the time of initial diagnosis to the date of death, and was calculated using the Kaplan–Meier method. mOS was calculated for each year separately, or for periods, as relevant for the presented analyses. Landmark analyses were conducted regarding two-year survival (2yS) and five-year survival (5yS). Since the current SEER data are updated till 2015, the most recent 5yS data were from patients diagnosed during 2010. To ensure that those who did not survive a full month after diagnosis were included in the analysis, patients coded in the SEER data set as having a survival time of zero were assigned a survival time of half a month. The effects of demographic, pathologic, and treatment variables on survival were tested with a Cox univariate analysis. Multivariate analysis was performed with a Cox proportional hazard model. All *p* values were 2 sided, and a *p* < 0.05 was considered statistically significant.

## 3. Results

### 3.1. Patient Characteristics

A consort diagram detailing the selected population is presented in Supplementary Figure 1. A total of 280,655 subjects with mNSCLC were included in the analysis; the median age was 67 years, and 58% were male. Demographic characteristics changed significantly from 1973 to 2015 (summarized by decades in Supplementary Table 1): the percentage of females increased from 25% in 1973 to 46% in 2015, median age increased from 62 to 68 years, proportion of white subjects decreased (from about 86% to 78%), and proportion of married decreased from 73% to 51% (all statistically significant; supplementary Table 1). The median income in 2010 at the county of residence of each patient decreased throughout the study period, probably reflecting the addition of counties with a lower median income to the SEER database.

### 3.2. Changes in Overall Survival

**Figure 1:**
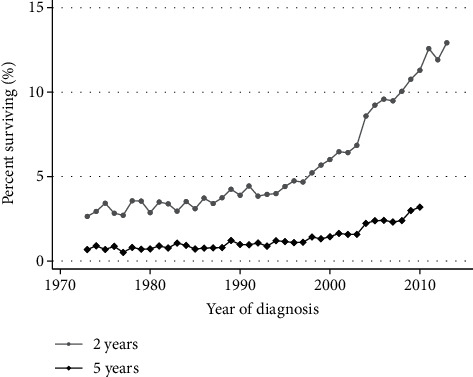
Changes in overall survival of metastatic lung cancer patients, 1973–2015. Trends in proportion of patients surviving 2 and 5 years, as a function of year of diagnosis. Both two- and five-year overall survival have improved over time (both *p* < 0.001, extended Wilcoxon rank-sum test).

The mOS for the entire cohort (*N* = 280,655) was 4 months (survival data in the SEER database are rounded to full months). Over time, the mOS has improved from two months in 1973 to five months in 2015. Regarding long-term survival, a clear rise in 2yS is noted, increasing from 2.6% in 1973 to 12.9% in 2013 (latest year of which 2yS data can be calculated; Figure 1 and Tables 1 and 2), occurring mostly after the mid-1990s. A more modest increase is seen in the 5yS, from 0.7% in 1973 to 3.2% in 2010 (latest year of which 5yS data can be calculated), also seen mostly after the mid-1990s.

### 3.3. Demographic Factors' Impact on Survival

**Figure 2:**
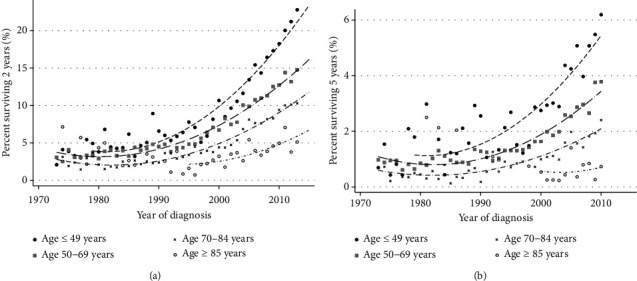
(a) Changes in the proportion of metastatic lung cancer patients surviving two years as a function of age and year of diagnosis. The symbols represent data for a specific year, and the lines indicate a trend line. (b) Changes in the proportion of metastatic lung cancer patients surviving five years as a function of age and year of diagnosis. The symbols represent data for a specific year, and the lines indicate a trend line.

A clear survival advantage was seen in favor of the younger patients (Tables 1 and 2). For instance, amongst patients of 49 years or below, the 2yS has increased from 2.1% to 22.8% (Figure 2) and the 5yS has increased from 0.7% to 6.2%. Proportionally similar rises can be seen in the older age groups and are significant for the 2yS; however, the numerical improvements in the 5yS are minimal. Regarding sex, improvements in both 2yS and 5yS have been more profound amongst women (Supplementary Figure 2). Married people have a better survival than unmarried, and the difference and the improvement with time are seen mostly in the 2yS (Supplementary Figure 3).

Race was a significant prognostic factor, with black patients demonstrating a better 2yS than white patients and Asian or Pacific Islanders showing better 2yS and 5yS compared to white and black patients (Tables 1 and 2). Asian/Pacific Islanders had the clearest advantage compared to whites. The numbers of American Indian or Alaska Native patients were small in earlier periods, resulting in highly unstable survival rates when comparing different years. Therefore, only data from 2000 onwards are presented for this group of patients (Supplementary Figure 4, left lower panel).Over time, improvement in outcome is seen amongst all ethnic groups in 2yS. However, only for the Asian/Pacific Islanders, the 5yS gets to 5% (5.2% for the 2010 cohort, 95% CI 3.9–6.8). The group of ‘unknown/other' race demonstrated better outcome but will not be discussed further due to the small number of patients.

### 3.4. Pathologic Factors

**Figure 3:**
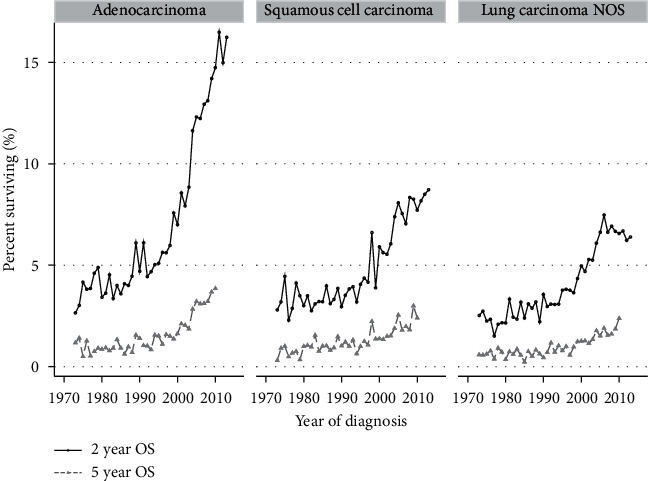
Trends in two-year and five-year survival 1973–2015 in different histologic subgroups. Yearly data are presented for each group.

We noted an increase in adenocarcinomas (from 29% in 1973 to 66% in 2015, *p* < 0.001) and a decrease in the proportion with squamous cell lung cancers (from 27.2% to 19.8%, *p* < 0.001). There was no consistent trend of change regarding the proportion of patients with non-other specified (NOS) NSCLC or with SCLC. Regarding outcome, the improvement in 2yS is the most noticeable in the adenocarcinoma subgroup of patients, from 1995 and onward (Figure 3). However, the 5yS remains below 5% for all histologic groups.

Aiming to identify subgroups with better outcome in an exploratory manner, we combined some of the favorable histologic and demographic factors. Females younger than 50 years with adenocarcinoma diagnosed from 2000 onwards had a 5yS of 6.8% (95% CI 6.0–7.8; number at risk at five years: 172).

Pathological-grade data were missing for 58% of the study cohort (these patients were, nonetheless, included in the analysis of other covariates). Among the 121,583 patients with grade data, a significant impact of this factor can be demonstrated, with 5yS of 6.3% for the well-differentiated tumors subgroup considering the entire study cohort (i.e., years of diagnosis: 1973 till 2010; Table 2). In patients with well-differentiated tumors, the 2yS reached 30.5% and 5yS reached 10.6% at the end of the analyzed period (95% CI 7.4–14.4).

### 3.5. The Role of Treatment Modalities

**Figure 4:**
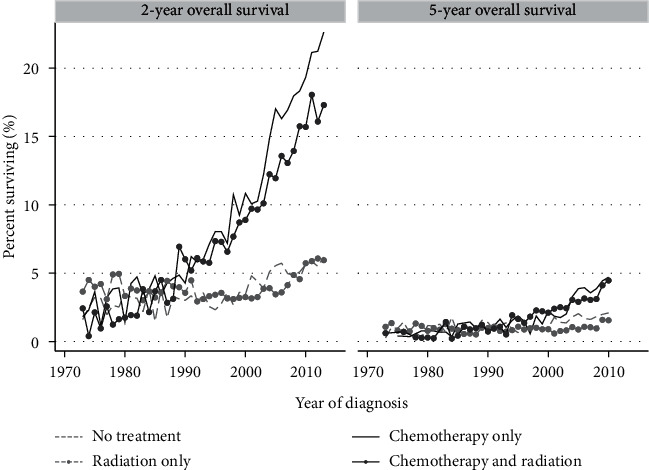
Proportion of metastatic lung cancer patients surviving two or five years according to treatment modality. Yearly data are presented for each subgroup, separated by the designated treatment modalities.

Data about treatments entered in the SEER database include general chemotherapy and radiotherapy categories. The proportion of patients listed as receiving chemotherapy increased from 28% in 1973 to 52% in 2015 (Supplementary Table 1). As can be seen in Figure 4, the improvement in 2yS is significant in patients that received either chemotherapy or combined chemotherapy and radiotherapy. 5yS also improved, but at best reaches 4.6% (95% CI 4.1–5.1).The groups that received only radiotherapy had similar low outcomes as those receiving no therapy. We were unable to demonstrate that any particular histologic subtype especially benefited from chemotherapy.

## 4. Discussion

We have described the 2yS and 5yS of lung cancer patients over a period of 43 years, with improvement seen mostly in the later fifteen years. Better outcomes are most pronounced among younger, married patients (Figure 2 and Supplementary Figure 3), in those bearing adenocarcinoma histology (Figure 3) and lower-grade tumors. Better outcome is seen in patients that have received chemotherapy (Figure 4). Race impacted survival, with a generally better outcome for nonwhite patients. The latest years analyzed here were 2013 regarding 2yS and 2010 regarding 5yS, before immunotherapy entered the arena of lung cancer treatments. Another large study from the preimmunotherapy period, of the International Association for the Study of Lung Cancer (IASLC), summarized world-wide data of more than 90,000 lung cancer patients diagnosed between 1999 and 2010. In that report, 2yS of 23% is reported for stage IVA and 6% for stage IVB and 5yS of 10% of stage IVA and 0% for stage IVB (clinical stage according the 8^th^ staging edition) [27]. The distinction between stage IVA and IVB is not available within the SEER database. However, our general conclusion that even among mNSCLC patients, specific subgroups have demonstrated nonnegligible long-term survival during the preimmunotherapy period is supported by the IASCLC study. Recently, Howlader et al. linked death certificate data to the SEER database in order to study changes in NSCLC incidence and mortality over the period 2001–2016, and their study was not confined to metastatic disease. Similar to our findings, they demonstrated marked improvements in 2-year mortality amongst both men and women [28].

The subgroup that stands out with a long 5yS is the younger patients, with age being a significant prognostic factor, as reported previously [29, 30]. Among the younger patients, the improvement in outcome over the years is the most striking. Multiple factors probably contribute to this phenomenon, including better performance status, less comorbidities, and a higher rate of targetable mutations. The improvement in the younger patients is seen starting from the mid-90s, similar to the rest of our cohort, prior to the addition of the targeted agents to the treatment options.

Race is well known to correlate with cancer incidence and outcome [19]. Regarding 5yS, black patients had a similar outcome to white patients in our data, as reported in other publications [29]. A markedly better outcome was found in our study for Asian or Pacific Islanders, as reported earlier [31]. The group of ‘Asian or Pacific Islanders' has been reported to have a relatively low incidence of lung cancer [32], comprising 6.7% of all lung cancer patients in our cohort. This is a complex group consisting, among others, of patients originated from eastern Asia, possibly including a higher proportion of EGFR mutation-positive patients. However, as discussed for the younger patients and as seen for the entire cohort, the improvement can be seen prior to the emergence of EGFR inhibitors or other targeted agents. Our findings resonate with other observations suggesting better outcome of Asian lung cancer patients compared to patients from the Western Hemisphere [33].

A recurrent observation is the prognostic value of socioeconomic status [34, 35] and, more interestingly, of marital status. Marital status has been found to predict lung cancer survival for NSCLC [35], as well as SCLC [36], although not in all reports [29]. Being married correlates with better survival for cancer patients in general [37], with diverse possible explanations, including better social support systems for married patients and better socioeconomic status, as well as selection bias; individuals who marry are more likely to be healthier than those that do not marry [38].The impact of being married, as well as the significant impact of the patients' social status, stresses the importance of providing comprehensive support to cancer patients, above and beyond the provision of the medical care.

A significant and strong prognostic factor in our cohort is the pathologic grade of the tumor, as reported previously in smaller data sets [30, 39, 40]. Tumor grade was available only for 43% of the study cohort, thus reducing the validity of this observation. Lack of clear definitions for pathologic grading of lung cancer impedes the inclusion of this important factor in a standardized manner in pathologic reports and in the staging system. It should be noted that only 5% of the patients in our cohort had well-differentiated tumors, and even moderately differentiated tumors constituted only 20.6% of the cohort. However, the better differentiated tumors have a markedly better prognosis. We, therefore, suggest that grade should be clearly identified in routine pathologic assessments of lung cancer specimens, as well as in clinical trials.

The reasons for the OS improvement seen along the years are most likely multifactorial. Conceivably, some of the improvement are the result of improved systemic treatments. This is supported by the correlation between chemotherapy treatments and improved outcome along the years (Figure 4). Clearly a selection bias exists in this analysis, as chemotherapy administration is usually limited to patients with relatively good performance status, a recognized prognostic factor by itself [2]. Regarding NSCLC, the use of chemotherapy vs. supportive care alone gradually gained support during the 1990s [15], which is also the period when the survival curves start to rise. Tailoring of chemotherapy drugs to tumor histology [6] and addition of anti-VEGF for nonsquamous NSCLC [41] are the major changes in chemotherapy choices that entered practice during 2000–2010; each of these had been demonstrated to improve median survival of clinical trial patients.

The next breakthrough in the care of NSCLC was targeted agents, initially the drugs targeting the EGFR receptor. Since the entrance of targeted agents, survival has extended for patients harboring the relevant genetic aberrations. Young, female, and adenocarcinoma are the groups where most of the improvement was seen in our cohort, and these are also the characteristics of patients harboring most of the driver mutations [42, 43]. The predictive power of EGFR mutations (representing 17% of the adenocarcinoma patients [44]) was discovered in 2004 [45, 46]. However, the EGFR mutation testing and appropriate therapy entered practice only in 2009 [4], thus, may be, affecting our latest 2yS data and minimally the most recent 5yS data. ALK rearrangement occurs in only 3–5% of NSCLC [47] and, hence, unlikely to impact the survival results of our study cohort. Importantly, the identification of tumors bearing ALK translocations as candidates for ALK inhibitor therapy occurred in 2010 [48], and the FDA approval of crizotinib for this indication occurred in 2011.

An additional potential explanation for improved mOS along the years is an overall more aggressive, less nihilistic approach to metastatic lung cancer, with a higher rate of patients receiving chemotherapy. A special example of aggressive therapy is the approach to oligometastatic disease [49, 50] with the use of locally ablative strategies, whether surgical, radiosurgical, or other. However, it should be noted that, when looking at the entire population, no correlation was found in our study between the use of radiotherapy and improved survival. Another relevant and related improvement that occurred along the years is the development of supportive care, recently demonstrated to prolong survival of lung cancer patients [7, 51].As a result of improved supportive care, more aggressive treatments can be administered to less robust patients, potentially improving survival of some of these patients.

The increase of mOS might be related also to Will Roger's phenomenon, i.e., the increased likelihood with time to correctly classify minimally metastatic patients, whereas in the past such tumors may have been understaged. Such improved staging would include metastatic patients with a low burden of disease within the metastatic patient cohort, thus improving the median survival of the cohort. The increased use of CT-PET, mediastinoscopy, and brain MRI for staging of patients [52] is likely to have an impact on the overall results of this study.

The strengths of our study include the large size of the database and the inclusion of patients excluded from clinical trials due to comorbidities and/or poor performance status. Furthermore, this is a well-validated and reliable data set with long-term follow-up [13, 53]. The shortcomings of our study include its retrospective nature, lack of basic clinical data such as performance status and weight loss, lack of data regarding molecular subtypes (especially EGFR mutation status and other driver mutations), and lack of details regarding systemic treatments administered. In addition, only a surrogate for socioeconomic status was captured, probably misclassifying many patients regarding this important prognostic parameter. Methods of staging have changed along the years as noted earlier, and these data are not captured in our data. As for any retrospective study, we cannot assign causative roles to the prognostic factors we have identified.

## 5. Conclusions

We have demonstrated substantial improvements over the last decades in the long-term survival of patients with metastatic lung cancer, mostly since the mid-90s. Most significant improvements were seen in younger, married patients, females, adenocarcinoma, low-grade tumors, and those receiving chemotherapy. While 2yS has bypassed 10% in recent years, 5yS is still in the range of 3–4%, stressing the need for further progress. Importantly, even prior to the era of immunotherapy and mostly before the emergence of targeted agents, subgroupsm NSCLC patients had long-term survival. The characteristics of these patients point to important prognostic factors, details of which should be collected and reported in current clinical trials.

## Figures and Tables

**Table 1:** Univariate and multivariate analysis of variables associated with overall survival in metastatic lung cancer patients, 1973–2015, Cox model. The multivariate analysis incorporated following variables: age, sex, race, marital status, histology, grade, year of diagnosis, treatment modality (of note regarding treatment, for each modality the database doses not differentiate between ‘treatment not given' and ‘unknown whether given'), and income (relating to county median income, 2010, in 10,000$). Patients with missing data for any one of these covariates were excluded from the multivariate analysis.

Variable^a^	Univariate analysis			Multivariate analysis		
	HR	95% C.I.	*p* value	HR	95% C.I.	*p* value
Age	1.013	(1.013, 1.013)	<0.001	1.007	(1.006, 1.008)	<0.001
Sex (female vs. male)	0.852	(0.839, 0.852)	<0.001	0.840	(0.825, 0.849)	<0.001

*Race*						
White	1.000 (comparator group)					
Black	0.989	(0.978, 1.001)	0.063	0.979	(0.960, 0.999)	0.045
Am. Indian/Alaska	1.011	(0.952, 1.074)	NS (0.72)	1.143	(0.986, 1.325)	0.076
Asian/Pacific Islander	0.753	(0.742, 0.765)	<0.001	0.832	(0.808, 0.856)	<0.001
Unknown/other	0.602	(0.524, 0.693)	<0.001	0.638	(0.475, 0.858)	0.003
Married	0.880	(0.873, 0.887)	<0.001	0.906	(0.893, 0.919)	<0.001

*Histology*						
Lung carcinoma NOS	1.000 (comparator group)					
Adenocarcinoma	0.736	(0.729, 0.742)	<0.001	0.860	(0.844, 0.875)	<0.001
Squamous cell ca.	0.866	(0.856, 0.875)	<0.001	0.910	(0.892, 0.929)	<0.001
Small cell carcinoma	0.858	(0.849, 0.866)	<0.001	1.029	(1.008, 1.05)	0.007

*Grade*						
Well diff.	1.000 (comparator group)					
Moderately diff.	1.173	(1.138, 1.209)	<0.001	1.190	(1.149, 1.233)	<0.001
Poorly diff.	1.512	(1.470, 1.554)	<0.001	1.484	(1.436, 1.535)	<0.001
Undifferentiated	1.781	(1.725, 1.840)	<0.001	1.484	(1.425, 1.545)	<0.001

*Year of diagnosis*						
1973–9	1.000 (comparator group)					
1980–9	0.948	(0.928, 0.968)	<0.001	0.952	(0.918, 0.987)	0.008
1990–9	0.906	(0.889, 0.924)	<0.001	0.935	(0.903, 0.969)	<0.001
2000–9	0.773	(0.759, 0.787)	<0.001	0.821	(0.793, 0.850)	<0.001
2010–5	0.663	(0.651, 0.676)	<0.001	0.739	(0.712, 0.766)	<0.001

*Treatment*						
No treatment	1.00 (comparator group)					
Chemotherapy only	0.423	(0.418, 0.428)	<0.001	0.488	(0.478, 0.498)	<0.001
Radiotherapy only	0.766	(0.759, 0.774)	<0.001	0.772	(0.758, 0.787)	<0.001
Chemo + radio	0.445	(0.440, 0.449)	<0.001	0.517	(0.507, 0.527)	<0.001
Income^b^	0.977	(0.974, 0.980)	<0.001	0.979	(0.974, 0.985)	<0.001

HR: hazard ratio. CI: confidence interval. Am: American. NOS: non-other specified. Ca: carcinoma. Diff: differentiated. Chemo: chemotherapy. Radio: radiotherapy. ^a^Missing values: grade: 207,149 patients. Marital status: 12,727 patients. Treatment: 10,881 patients. Age: 3 patients. Income: 73,156 patients. ^b^2010 yearly median county income (per 10,000 dollars).

**Table 2:** Percentage survival at two and five years by prognostic factors.

	2yOS		5yOS	
	%	95% CI	%	95% CI
Entire cohort	8.0	(7.9–8.2)	2.1	(2.0–2.1)

*Age*				
<67	9.5	(9.3–9.6)	2.7	(2.6–2.7)
≧67	6.6	(6.5–6.7)	1.5	(1.5–1.6)

*Sex*				
Male	6.3	(6.1–6.4)	1.6	(1.5–1.7)
Female	10.5	(10.3–10.7)	2.8	(2.7–2.9)

*Race*				
White	7.5	(7.4–7.7)	2	(1.9–2.0)
Black	7.2	(6.9–7.5)	1.8	(1.7–2.0)
American Indian/Alaska native	8.6	(7.0–10.4)	1.5	(0.8–2.6)
Asian/Pacific Islander	15.1	(14.5–15.6)	4.1	(3.7–4.4)
Unk/oth	23.1	(17.6–29.1)	15.1	(9.8–21.5)

*Marital status*				
Single	7.0	(6.9–7.2)	1.8	(1.7–1.9)
Married	8.7	(8.6–8.9)	2.3	(2.2–2.4)

*Histology*				
Adenocarcinoma	10.8	(10.6–11)	2.7	(2.6–2.8)
Lung carcinoma NOS	5.1	(5.0–5.3)	1.3	(1.3–1.4)
Squamous cell carcinoma	6.2	(6.0–6.4)	1.8	(1.7–1.9)
Small-cell carcinoma	4.4	(4.3–4.6)	1.3	(1.2–1.4)

*Grade*				
Well differentiated	18.6	(17.6–19.7)	6.3	(5.6–7.0)
Moderately differentiated	13.2	(12.8–13.7)	3.8	(3.5–4.1)
Poorly differentiated	7.1	(6.9–7.3)	2.0	(1.9–2.1)
Undifferentiated	4.5	(4.1–4.8)	1.3	(1.1–1.5)

*Year of diagnosis*				
1973–79	3.1	(2.9–3.4)	0.7	(0.6–0.9)
1980–89	3.5	(3.3–3.7)	0.9	(0.8–1.0)
1990–99	4.5	(4.3–4.7)	1.1	(1.0–1.2)
2000–09	8.4	(8.3–8.6)	2.1	(2.0–2.2)
2010–15	12.6	(12.3–12.9)	3.7	(3.5–3.9)

*Treatment*				
None	4.5	(4.3–4.6)	1.6	(1.5–1.7)
Chemotherapy only	14.9	(14.6–15.2)	3.3	(3.1–3.5)
Radiotherapy (RT) only	4.0	(3.9–4.2)	1.0	(1.0–1.1)
Chemotherapy + RT	11.7	(11.5–12)	2.9	(2.8–3.1)

*Income*				
<median^a^	7.2	(7.0–7.4)	1.9	(1.8–2.0)
≧median^a^	8.5	(8.4–8.7)	2.2	(2.1–2.3)

2yOS: two-year overall survival. 5yOS: five-year overall survival. CI: confidence interval. NOS: non-other specified. Ca: carcinoma. ^a^Annual county median in 2010 of $52,595.

## Data Availability

The raw data are freely available from the website of the NCI's Surveillance, Epidemiology, and End Results (SEER) Program https://seer.cancer.gov/mortality/.

## Supplementary Materials

Supplementary Materials Figure S1: consort diagram of patients participating in this study. SCLC: small-cell lung cancer; Figure S2: trends in two-year and five-year survival 1973–2015 in males and females. Yearly data are presented for each group; Figure S3: proportion of surviving patients at different time points, stratified by marital status. Yearly data are presented for each group; Figure S4: proportion of surviving patients at different time points, stratified by the ethnic group. Yearly data are presented for each group. The numbers of American Indian or Alaska Natives patients were small in earlier periods; thus, only data from 2000 onwards are presented for this group of patients; Table S1: patients characteristics of the entire study population and by decades (based on the year of diagnosis).
