# Supplementary material for: Long-Term Survival of Patients with Metastatic Non-Small-Cell Lung Cancer over Five Decades
Source: J Oncol. 2021 Jan 12;2021:7836264. doi: 10.1155/2021/7836264 (PMC7817269; doi:10.1155/2021/7836264)
Supplement: Supplementary Materials — Figure S1: consort diagram of patients participating in this study. SCLC: small-cell lung cancer; Figure S2: trends in two-year and five-year survival 1973–2015 in males and females. Yearly data are presented for each group; Figure S3: proportion of surviving patients at different time points, stratified by marital status. Yearly data are presented for each group; Figure S4: proportion of surviving patients at different time points, stratified by the ethnic group. Yearly data are presented for each group. The numbers of American Indian or Alaska Natives patients were small in earlier periods; thus, only data from 2000 onwards are presented for this group of patients; Table S1: patients characteristics of the entire study population and by decades (based on the year of diagnosis). [file 7836264.f1.docx]

**Supplementary Material:**

Long-term survival of patients with metastatic non-small-cell lung cancer prior to the immunotherapy era

**Figure S1:** Consort diagram of patients participating in this study.

SCLC: small cell lung cancer


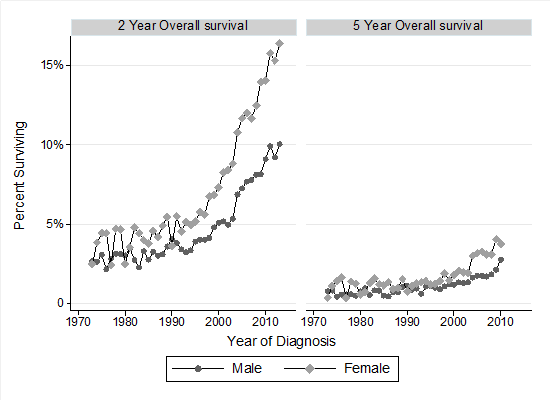


**Figure S2:** Trends in two and five-year survival 1973-2015 in males and females. Yearly data is presented for each group.
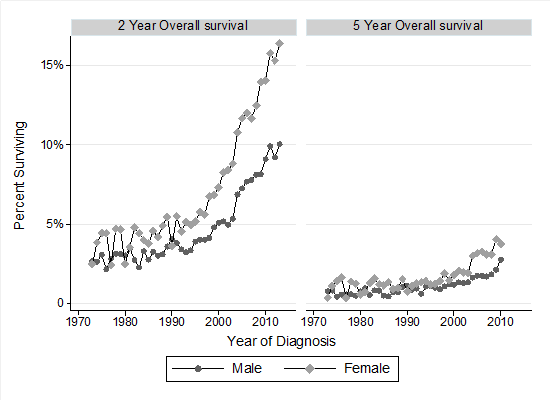


**Figure S3:** Proportion of surviving patients at different timepoints, stratified by marital status. Yearly data is presented for each group.
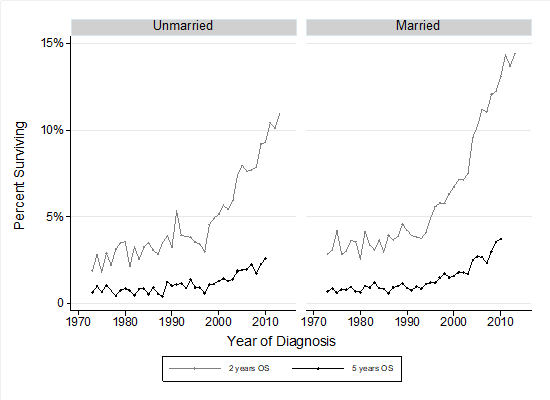


**Figure S4:** Proportion of surviving patients at different time points, stratified by ethnic group. Yearly data is presented for each group. The numbers of American Indian or Alaska Natives patients were small in earlier periods, thus only data from 2000 onwards is presented for this group of patients.


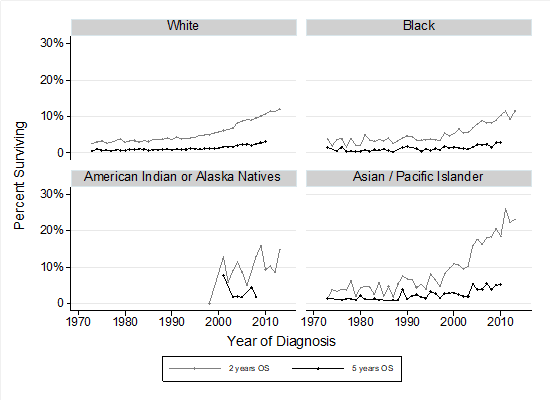


**Table S1: Patients characteristics of entire study population and by decades (based on year of diagnosis).**

|  | **Total** | | | **1973-79** | | | **1980-89** | | **1990-99** | | **2000-09** | | | **2010-15** | | **P value**^a^ |
| --- | --- | --- | --- | --- | --- | --- | --- | --- | --- | --- | --- | --- | --- | --- | --- | --- |
| **N,%** ^b^ | **280,655** | **100** | **13,561** | | **4.8** | **26,742** | | **9.5** | **43,631** | **15.5** | **121,100** | **43.1** | **75,621** | | **26.9** |  |
| **Age, years** | **Median** | **IQR(25-75%)** | **Median** | | **IQR(25-75%)** | **Median** | | **IQR(25-75%)** | **Median** | **IQR(25-75%)** | **Median** | **IQR(25-75%)** | **Median** | | **IQR(25-75%)** |  |
|  | **67** | **59-75** | **63** | | **55-71** | **65** | | **57-72** | **67** | **58-74** | **68** | **59-76** | **68** | | **60-76** | **<0.0001** |
|  | **N** | **%** | **N** | | **%** | **N** | | **%** | **N** | **%** | **N** | **%** | **N** | | **%** |  |
| **Male** | **164,191** | **58.5** | **9,886** | | **72.9** | **23,746** | | **66.5** | **33,232** | **59.1** | **86,150** | **56.0** | **52,589** | | **54.5** | **<0.0001** |
| **Ethnicity** | **N** | **%** | **N** | | **%** | **N** | | **%** | **N** | **%** | **N** | **%** | **N** | | **%** | **<0.0001** |
| **White** | **225,185** | **80.2** | **11,503** | | **84.8** | **22,694** | | **84.9** | **35,014** | **80.3** | **97,271** | **80.3** | **58,703** | | **77.6** |  |
| **Black** | **35,158** | **12.5** | **1,432** | | **10.6** | **2,990** | | **11.2** | **5,452** | **12.5** | **15,284** | **12.6** | **10,000** | | **13.2** |  |
| **American Indian/Alaska Native** | **1135** | **0.4** | **12** | | **0.1** | **37** | | **0.1** | **173** | **0.4** | **479** | **0.4** | **434** | | **0.6** |  |
| **Asian/Pacific Islander** | **18,883** | **6.7** | **604** | | **4.5** | **1,013** | | **3.8** | **2,965** | **6.8** | **7,969** | **6.6** | **6,332** | | **8.4** |  |
| **Unknown/Other** | **294** | **0.1** | **10** | | **0.1** | **8** | | **0.0** | **27** | **0.1** | **97** | **0.1** | **152** | | **0.2** |  |
| **Married** | **150,157** | **55.5** | **8,941** | | **67.3** | **16,626** | | **63.8** | **24,414** | **57.5** | **63,122** | **54.0** | **37,054** | | **51.4** | **<0.0001** |
| **Histology** | **N** | **%** | **N** | | **%** | **N** | | **%** | **N** | **%** | **N** | **%** | **N** | | **%** | **<0.0001** |
| **Adenocarcinoma** | **133,929** | **47.7** | **4683** | | **34.5** | **11021** | | **41.2** | **19607** | **44.9** | **52,955** | **43.7** | **45,663** | | **60.4** |  |
| **Squamous** | **53,260** | **19.0** | **3564** | | **26.3** | **6595** | | **24.7** | **8054** | **18.5** | **19851** | **16.4** | **15196** | | **20.1** |  |
| **NOS** | **93,466** | **33.3** | **5314** | | **39.2** | **9126** | | **34.1** | **15970** | **36.6** | **48294** | **39.9** | **14762** | | **19.5** |  |
| **Grade** | **N** | **%** | **N** | | **%** | **N** | | **%** | **N** | **%** | **N** | **%** | **N** | | **%** | **<0.0001** |
| **Well differentiated** | **5,849** | **4.8** | **275** | | **4.5** | **488** | | **3.3** | **810** | **3.5** | **2,509** | **5.2** | **1,767** | | **5.9** |  |
| **Moderately differentiated** | **24,986** | **20.6** | **593** | | **9.7** | **2,045** | | **13.8** | **3,687** | **16.0** | **10,441** | **21.8** | **8,220** | | **27.6** |  |
| **Poorly differentiated** | **77,758** | **64.0** | **2,925** | | **47.9** | **8,798** | | **59.5** | **15,393** | **66.7** | **31,660** | **66.1** | **18,982** | | **63.8** |  |
| **Undifferentiated** | **12,990** | **10.7** | **2,314** | | **37.9** | **3,455** | | **23.4** | **3,179** | **13.8** | **3,269** | **6.8** | **773** | | **2.6** |  |
| **Treatment** | **N** | **%** | **N** | | **%** | **N** | | **%** | **N** | **%** | **N** | **%** | **N** | | **%** | **<0.0001** |
| **No treatment** | **86,429** | **31.8** | **4,027** | | **30.3** | **6,778** | | **25.9** | **12,715** | **30.2** | **39,212** | **33.5** | **23,697** | | **32.3** |  |
| **Chemotherapy only** | **51,139** | **18.8** | **1,699** | | **12.8** | **3,098** | | **11.8** | **5,843** | **13.9** | **23,548** | **20.1** | **16,951** | | **23.1** |  |
| **Radiotherapy only** | **68,679** | **25.3** | **5,360** | | **40.3** | **11,704** | | **44.8** | **14,747** | **35.0** | **24,470** | **20.9** | **12,398** | | **16.9** |  |
| **Chemotherapy +**  **radiotherapy** | **65,713** | **24.2** | **2,203** | | **16.6** | **4,564** | | **17.5** | **8,782** | **20.9** | **29,836** | **25.5** | **20,328** | | **27.7** |  |
| **Income (county, 2010), US$** | **Median** | **IQR(25-75%)** | **Median** | | **IQR(25-75%)** | **Median** | | **IQR(25-75%)** | **Median** | **IQR(25-75%)** | **Median** | **IQR(25-75%)** | **Median** | | **IQR(25-75%)** |  |
|  | **52,595** | **42,690-60,392** | **57,070** | | **42,672-70,805** | **57,070** | | **45,083-66,937** | **52,595** | **49,348-66,076** | **52,595** | **42,535-59,759** | **52,595** | | **41,945-59,759** | **<0.0001** |

IQR: interquartile range. NOS: non-other specified. ^a^P value indicates statistical significance of change along the decades. ^b^Percentages relate to the total of each column, regarding the relevant category.
